# Protease-Activated Receptor 4 (PAR4)-Tethered Ligand Antagonists Demonstrate Thrombin Liability

**DOI:** 10.1021/acsptsci.5c00626

**Published:** 2025-12-23

**Authors:** Emma M. Webb, Jackson B. Cassada, Heidi E. Hamm

**Affiliations:** † Department of Pharmacology, 5718 Vanderbilt University, Nashville, Tennessee 37232-6600, United States; ‡ Department of Biochemistry, 5718 Vanderbilt University, Nashville, Tennessee 37232-6600, United States

**Keywords:** Pharmacology, Protease-activated Receptor 4 (PAR4), Thrombin, Proteases

## Abstract

The Hamm laboratory recently published a cohort of PAR4 antagonists that were effective against the tethered ligand activation of PAR4. These compounds were generated from an ultralarge virtual screen using a homology model of PAR4. Upon further investigation, it appears the protease-activated receptor antagonists highlighted in this work have some thrombin liability. The Hamm laboratory further characterized the activity of these compounds using various methods, including a fluorescent thrombin activity assay, a chromogenic thrombin activity assay, and flow cytometry assays. We conclude that they do indeed antagonize PAR4, but thrombin is an additional target.

## Graphical abstract

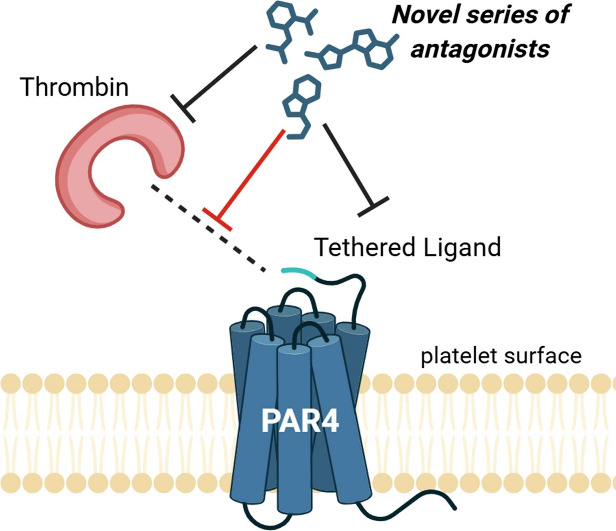

Protease-activated receptor 4 (PAR4) is a G protein coupled receptor (GPCR) most widely expressed on human platelets. It is a novel GPCR due to its tethered ligand mechanism of activation. Serine proteases, such as thrombin and trypsin, cleave the N-terminus of PAR4, revealing a new N-terminus that folds back and activates the receptor.^1,2^ With the endogenous ligand covalently attached to the receptor, the antagonism of PAR4 has always been difficult. Previous drug discovery efforts have been successful against the agonist peptide mimetic of the PAR4 tethered ligand sequence, but few have conquered the feat of antagonizing the endogenous tethered ligand.^3–8^

We recently published a cohort of PAR4 antagonists that were effective specifically against the tethered ligand.^9^ These compounds were generated from an ultralarge virtual screen using a homology model of PAR4. Through computational similarity searches and medicinal chemistry efforts, we generated compounds that improved from 2.3 uM to about 100 nM potency in human platelets.^9^ Here, we report an additional target for these PAR4 antagonists.

Upon further investigation, it appears the protease-activated receptor antagonists highlighted in this manuscript have some thrombin liability. We further characterized the activity of these compounds using various methods, including a fluorescent thrombin activity assay, a chromogenic thrombin activity assay, and flow cytometry assays. We conclude that they do indeed antagonize PAR4; however, thrombin is an additional target.

## Methods

### Fluorescent Thrombin Inhibition Assay

A Thrombin Inhibition Kit was ordered from Abcam (Cambridge, UK). It contained thrombin assay buffer, thrombin dilution buffer, thrombin inhibitor (PPACK), thrombin standard, and a fluorometric thrombin substrate. It was used according to the manufacturer’s instructions.

### Chromogenic Thrombin Activity Assay

Chromogenic substrate, S-2238, was ordered from DiaPharma (West Chester, OH). Alpha-thrombin was ordered from Enzyme Research Laboratories (South Bend, IN). White, round, clear-bottom 96-well plates were used to run the screen. The plate was read using a BioTek Neo plate reader every minute for 60 min at a wavelength of 405 nm. The substrate control wells were subtracted from the raw counts, and the data was normalized to the thrombin enzyme control wells as a percentage. The percentages were graphed in GraphPad Prism.

### Human Platelet Flow Cytometry Assay

Same as described previously.^9^

## Results and Discussion

To investigate thrombin inhibition, we utilized a chromogenic assay with a thrombin substrate, S-2238. Thrombin activity was measured by the production of a visible color change to substrate S-2238. Each assay run included a substrate control, where just substrate was added to the well without thrombin enzyme, an enzyme control (EC), which consisted of thrombin without any inhibitor present, and an inhibitor control (IC), which contained a known thrombin inhibitor, Argatroban. Figure 1 demonstrates the results of an *N* = 2 of a robust screen of compounds from the virtual screen and includes compounds from each scaffold generation. Each compound was screened at 500 nM against 10 nM alpha thrombin. The compounds exhibited a wide range of thrombin inhibition, with some of the most potent compounds, 31 and 12, exhibiting about 70% inhibition of thrombin.

**Figure 1:**
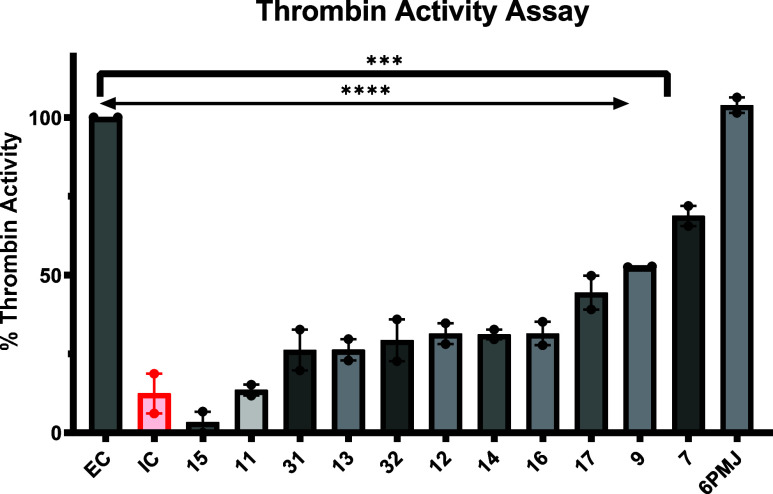
Thrombin inhibition of PAR4 tool compounds (500 nM) utilizing a chromogenic assay to capture thrombin activity. Mean + SEM, *N* = 2; one-way ANOVA, Dunnett’s post hoc show a significant difference in absorbance between enzyme control (EC) and all compounds (*P* < 0.0001 ****; *P* < 0.0005 ***) except 6PMJ.

To also characterize what type of inhibition was occurring with these compounds, we performed Michaelis–Menten enzyme kinetic analysis. The Lineweaver–Burk plot shows that the compounds are direct thrombin inhibitors, as the Vmax did not shift but the Km did (Figure 2 and Table 1). This is not a surprising finding, as thrombin is believed to interact with PAR4 only at the active cleft of thrombin.^10^ PAR4 lacks the hirudin-like binding domain that PAR1 has, which enables PAR1 to have a higher affinity to thrombin.

**Figure 2:**
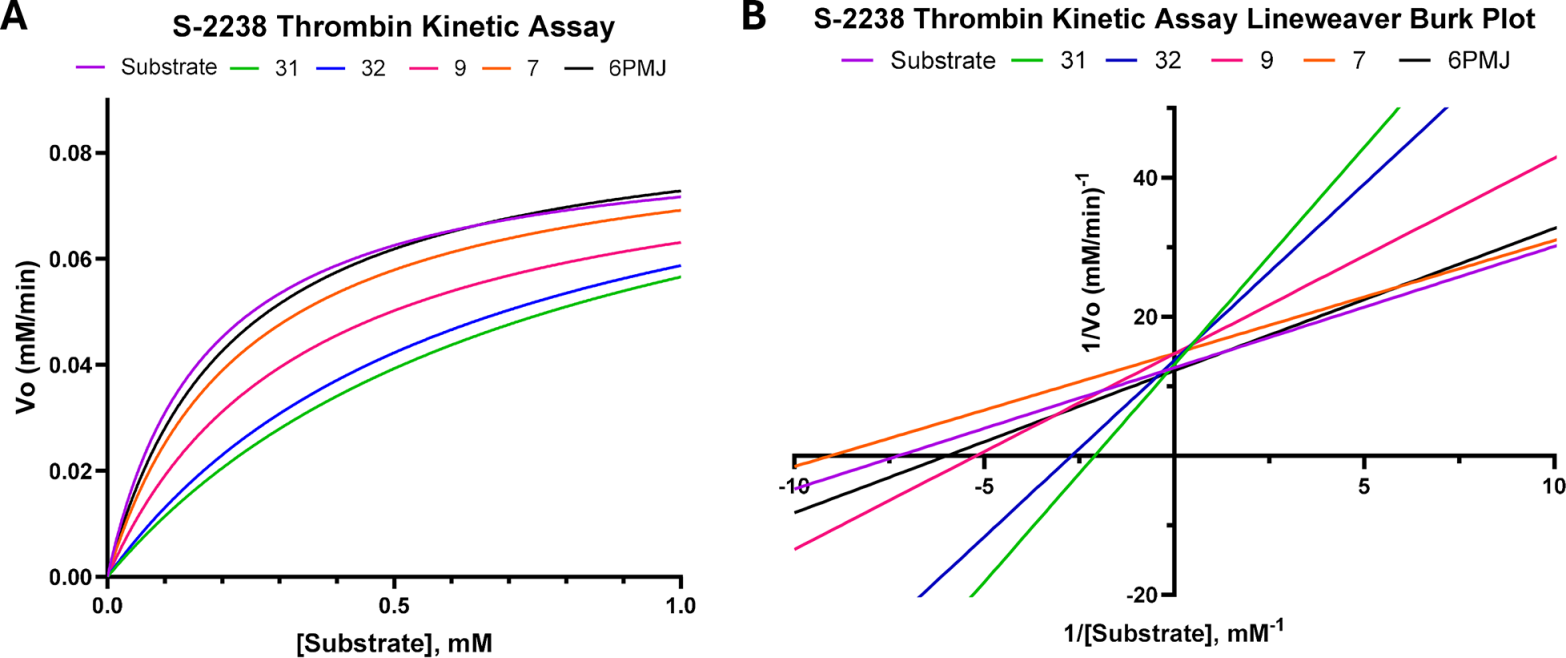
Thrombin inhibition characterization of PAR4 antagonists. A) Michaelis–Menten plot depicting thrombin enzymatic activity in the presence of various PAR4 antagonists. B) Lineweaver–Burk plot of the Michaelis–Menten plot.

**Table 1:**
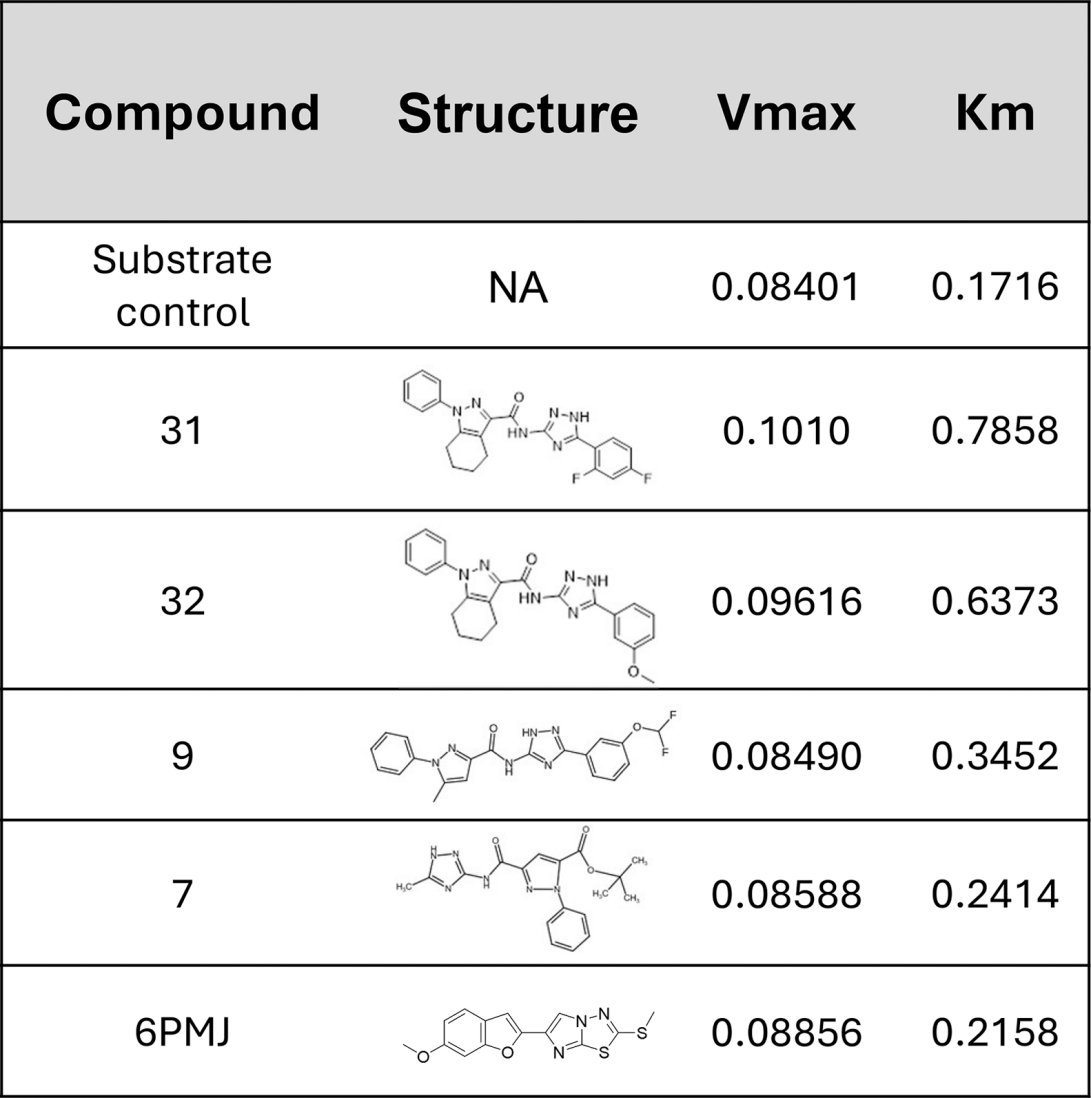
Enzyme Kinetics of Thrombin in the Presence of PAR4 Antagonists

The IC50s against thrombin were determined for representative compounds to compare to the previously obtained IC50s from flow cytometry experiments (Figure 3). In the flow cytometry experiments, thrombin activates PAR4 on human platelets. The IC50s between these two assays do not match those of either representative compound. Compound 12 had an IC50 of ∼71 nM in the thrombin activity assay and a PAR4 IC50 of ∼149 nM in the platelet assay (Figure 3A). Compound 31 had an IC50 of ∼1 uM in the thrombin activity assay and a PAR4 IC50 of ∼160 nM in the platelet assay (Figure 3B). In the flow cytometry assay, human platelets are stimulated with thrombin in the presence of either a PAR4 antagonist (BMS-3) or a PAR1 antagonist (Vorapaxar). This determines the IC50 of the test compound against PAR1 and PAR4 activation. The compounds generated from the virtual screen are more potent against PAR4 than against PAR1. In comparison, Argatroban, a potent thrombin inhibitor, demonstrates no preference for PAR4 over PAR1 in the same assay. Hirugen, a synthetic, hirudin-derived thrombin inhibitor, also demonstrates no preference for PAR4 over PAR1 in the alpha-thrombin flow cytometry assay (Figure 4). The functional results from platelet activity and thrombin activity assays indicate a functional preference for PAR4 over PAR1, with concurrent thrombin inhibition. These compounds have a dual modality of antagonizing PAR4 and inhibiting thrombin.

**Figure 3:**
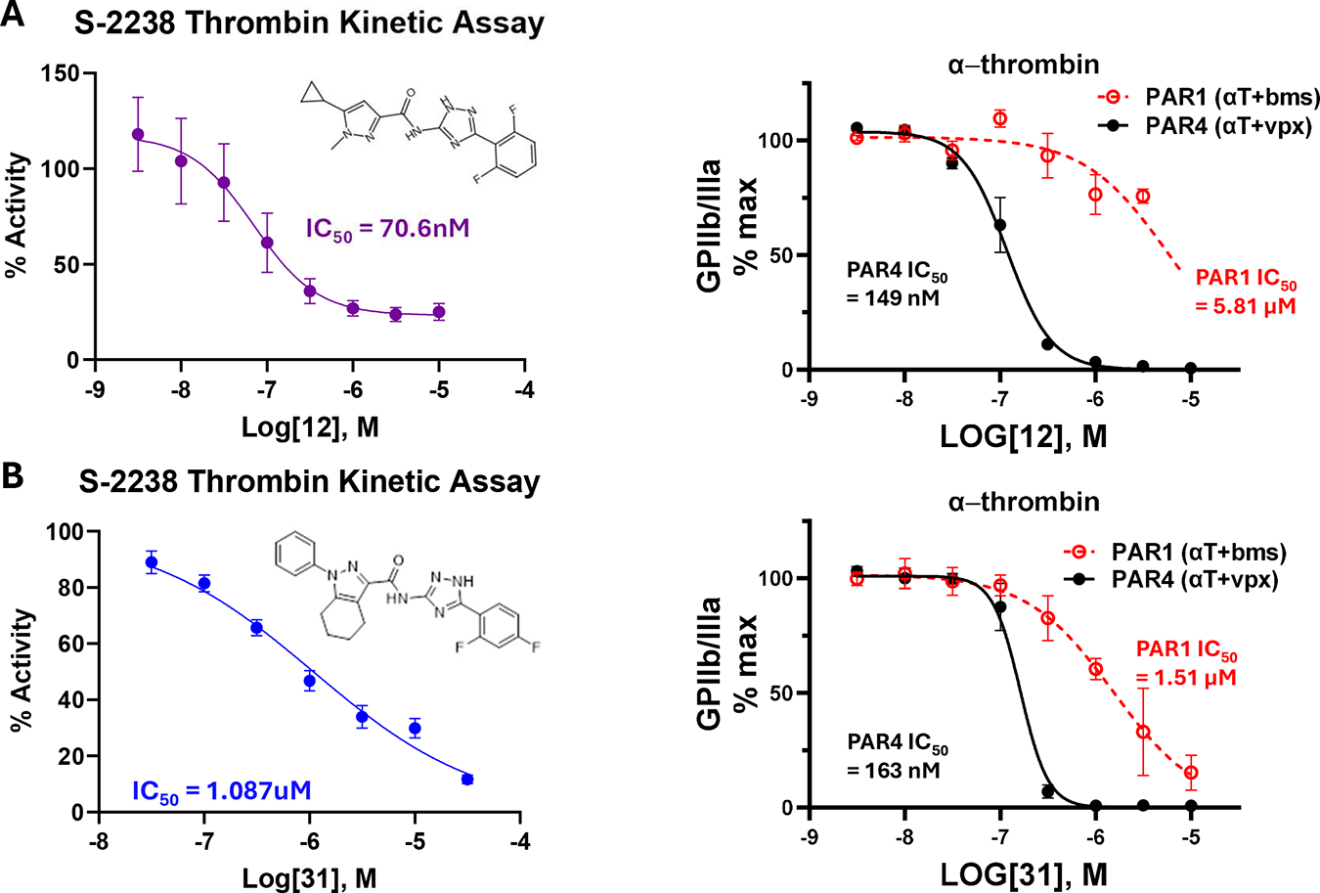
Concentration response curves differ for compounds in the thrombin activity assay and platelet activation assay. A) Compound 12 demonstrated an IC50 of 60.9 nM against thrombin and an IC50 of 149 nM against PAR4. B) Compound 31 demonstrated an IC50 of 1.087 uM against thrombin and an IC50 of 163 nM against PAR4. Mean + SEM, *N* = 3.

**Figure 4:**
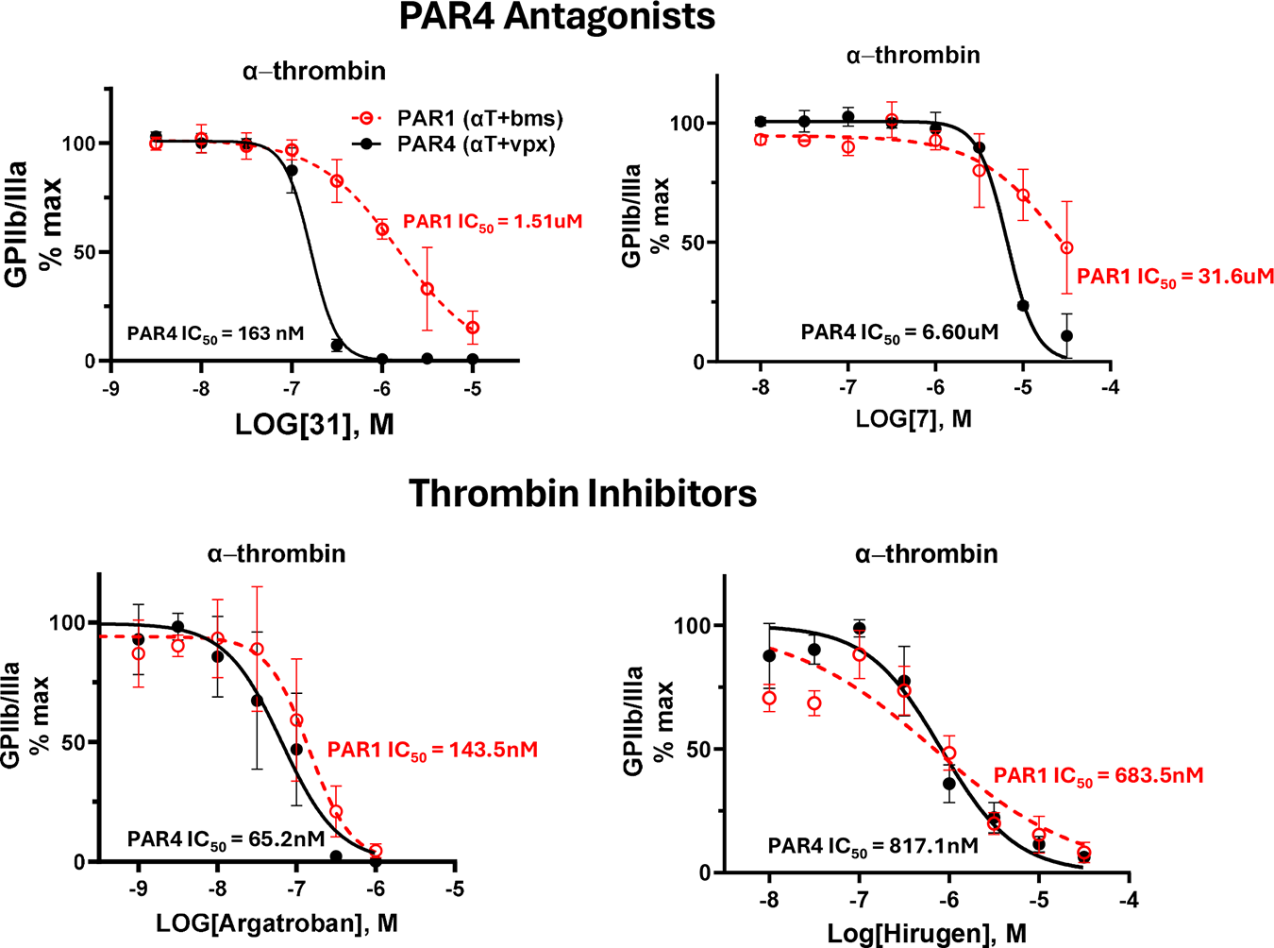
PAR4 antagonists exhibit PAR4 preference over PAR1 in the platelet assay probing α-thrombin activation: PAR1 (α-thrombin + BMS-3) vs PAR4 (α-thrombin + vorapaxar). Direct thrombin inhibitors exhibit no preference between PAR1 and PAR4 in the platelet assay. Mean + SEM, *N* = 3.

These compounds may prohibit the binding of thrombin with PAR4, preventing the activation of the receptor by the enzyme. To further characterize the dual modality of inhibition, further investigation into how these compounds are binding to PAR4 and thrombin are ongoing. The Hamm laboratory is concurrently investigating the binding interaction of thrombin to the N-terminus of PAR4 and if these compounds prohibit the interaction. These studies are ongoing and will be part of a larger paper dissecting the interactions of PAR4 and its known proteases.

## Notes

Informed consent was obtained from all healthy volunteers, in accordance with IRB #130814, for the human platelet assays.
